# The Pathology of Leucorrhœa

**Published:** 1855-08

**Authors:** 


The pathology of Leucorrhœa, by W. Tyler Smith, M. D., &c., &c., Philadelphia; Blanchard & Lea, 1855.

The writings of Dr. Smith, on this subject have already made the profession aware of the manner in which he has investigated this subject, and in part at least with the views which he entertains in regard to it—A careful antaomical and physiological examination of the structures implicated, has furnished a basis for a division of Leucorrhœa into two varieties, mucous and epithelial. The grounds of this division will, perhaps be better understood from the following extract. :

The demonstration of two very differently organized surfaces in the vagina, and in the canal of the cervix uteri, with the existence of two very distinct forms of leucorrhœa. But at this point it may be well to revert for a moment to the special differences which exist between the vagina and the cervical canal. The lining membrane of the vagina approaches in organization to the skin; it is covered by a thick layer of scaly epithelium; it contains in the greater part of its surface few if any mucous follicles or glands; its secretion is acid, consisting entirely of plasma and epithelium, and the chief object of the secretion is the lubrication of the surface upon which it is formed. On the other hand, the lining of the canal of the cervix is a true mucous membrane; it is covered in great part by cylinder epithelium; it abounds with immense numbers of mucous follicles having a special arrangement; it pours forth a true mucous secretion, alkaline in character, and consisting of mucus-corpuscles and plasma, with little or no epithelium; and this secretion has special uses to perform in the unimpregnated state, and in pregnancy and parturition. Leucorrhœa admits of a similar division. The first and the most frequent and important is the Mucous variety consisting chiefly of mucus-corpuscles and plasma, and secreted chiefly by the follicular canal of the cervix. The second is the Epithelial variety, in which the discharge is Vaginal, or is secreted by the vaginal portion of the os and cervix, and consists for the most part of scaly epithelium and its *debris*. These two varieties may of course exist in various degrees of combination; sometimes the one and sometimes the other preponderates, or is the original affection; but the chief importance must be given to cervical or mucous leucorrhœa, as being the most obstinate and common.

It has been shown by Dr. Smith that a mere physical examination, without the aid of the miscroscope, is not sufficient to determine the variety, although the secretion from the cervix is alkaline and glairy, and that of the vagina acid and creamy. The cervical discharge coming in contact with the vaginal mucous membrane, loses its characteristic appearance, and can only be distinguished by the microscope from the epithelial secretion of the surface with which it is in contact. The microscopic characters are contrasted as follows:

The following are the elements found in the discharges in vaginal or epithelial leucorrhœa of different degrees of severity—

1. Acid Plasma.
2. Scaly Epithelium.
3. Pus-Corpuscles.
4. Blood-globules.
5. Fatty Matter.

The following are the elements found in the different forms of cervical or mucous leucorrhœa—

1. Alkaline Plasma.
2. Mucus-Corpuscles.
3. Altered Cylinder Epithelium.
4. Pus-Corpuscles
5. Blood-Globules.
6. Fatty Particles.

The sequelæ of Leucorrhœa are discussed at some length.—These are inflammation, abrasion, ulceration, induration and hypertrohpy of the os and cervix uteri, and abrasion and superficial ulceration of the vagina. We quote the paragraph on the points of diagnosis between leucorrhœa and cancer. In addition to the cancer smell, he says—

To the naked eye, the commencement of carcinoma of the uterus can sometimes scarcely if at all be distinguished from common induration. Common induration may be quite as solid and stonelike to the touch as the cancerous hardness, but the schirrous affection often extends from the os uteri to the structure before and behind it, so that the os uteri becomes fixed, whereas this never happens in cases of simple induration, except in cases where there has been ulceration and cicitrization of the vagina. If it should happen that an incision be made into the induration, there is no longer any doubt, as the gritty secretion conveyed by schirrous uteri is altogether unlike that of simple ulceration. The age of the patient is of some value in the diagnosis, as common induration frequently occurs in young childbearing women, while schirrus is more common, as the catemenial decline approaches.

When cancerous induration begins to soften, especially if this process take place slowly, the granulations are frequently red, even and with a tolerably clear and well-defined margin. To the naked eye alone, the difference between malignant and common ulcerations is sometimes inappreciable. As soon, however, as ulceration commences, digital examination readily settles any doubt. The ulceration in cases of carcinoma is always rough, indurated, and harsh to the touch, while common ulceration is as constantly soft and yielding to the finger. If the speculum alone be trusted to, it is easy to mistake carcinomatous ulceration for simple ulceration, and *vice versa.* A short time since I had a case under my care at St Mary's Hospital, sent to me by Mr. Truran, of Truro, in which the naked-eye appearances were very deceptive. The case was considered to be one of corroding ulcer, but at the time she was admitted I happened to have a scratch upon the index finger, and examined her in consequence with the speculum only; the ulceration of the os and external surface was so smooth, and the natural shape of the os and cervix so well preserved, that I hesitated about considering it as carcinomatous. A few days afterwards, when I examined her digitally, there was no question about the the matter, but the information conveyed by sight and touch is sometimes, as in this case, quite contradictory, and therefore visual examination cannot alone be depended on. On the other hand, I have known simple ulceration present such an appearance to the eye as to lead to the strong suspicion of cancer. but the slightest touch of the finger has been sufficient to dispel the alarm. The occurrence of hemorrhage is of some value in a diagnostic point of view. Hemorrhage occurs in simple ulceration, but the loss is hardly ever so sudden and of such an extent as often occurs in cancer.

The examination of the discharge by the miscroscope does not invariably give satisfactory evidence in cases of carcinoma, but generally the heterogeneous compound cells, and the ill-formed cancer pus-cells are present, mixed up with enormous quantities of epithelium.

He next discusses the relations between secondary syphilis and leucorrhœa. The author thinks that syphilitic leucorrhœa occurs with certainty only under two conditions:

"1st. When the patient has contracted primary syphilis, and the secondary or constitutional affection has followed in due course.

2d. When the mother, being in health, is impregnated by a husband who is at the time affected with secondary syphilis, and receives the secondary syphilitic disorder through the medium of the ovum."

In chapter eighth the author discusses the relation of vaginal or epithelial leucorrhœa to gonorrhœa in the female, to urethritis in the male, and to opthalmia of new-born infants. In reference to urethritis in the male caused by leucorrhœa, the author says:

Cases are sometimes met with in practice, in which all the symptoms of gonorrhœa occur in the male, after intercourse with a woman affected with leucorrhœa only. No doubt in some of these cases, gonorrhœa may have existed in the female, but they occur so often as to leave, I think, little question that under certain circumstances, a spotaneous leucorrhœa arising independently of sexual intercourse, may produce urethritis and inflammation of the glans penis in the male. The urethritis thus caused can scarcely, if at all, be distinguished from gonorrhœa, the result of infection.

We have not time to follow the author further, although the remaining subjects are of much interest

Dr. Smith has done the profession a valuable service in the production of this little volume. That portion devoted to the treatment of leucorrhœa, especially supplies a want which the profession will no doubt appreciate.

The work is for sale by Keen & Lee. J.

